# Supplementary material for: Utilization of Cobalamin Is Ubiquitous in Early-Branching Fungal Phyla
Source: Genome Biol Evol. 2021 Mar 3;13(4):evab043. doi: 10.1093/gbe/evab043 (PMC8085122; doi:10.1093/gbe/evab043)
Supplement: evab043_Supplementary_Data [file evab043_supplementary_data.zip › Supplementary Legends.docx]

## Supplementary Legends

**Supplementary Table S1** Table with numbers of each eight cobalamin-dependent enzymes homologs in all studied non-Dikarya fungi.

**Supplementary Table S2** Accessions of all identified protein homologs.

**Supplementary Table S3** PMIDs of sources of information about all studied genomes.

**Supplementary Dataset DS1** File with phylogenetic trees of B12-dependent enzymes in non-Dikarya fungi in Newick format. Contains ML trees with all found homologs (full) and chosen subtree and BA subtree of all studied enzymes.
